# Impact of Water Sediment Quality on Germination of Submerged Aquatic Plants in Flemish Streams

**DOI:** 10.3390/plants14213290

**Published:** 2025-10-28

**Authors:** Lucas Van der Cruysse, Andrée De Cock, Pieter Boets, Peter L. M. Goethals

**Affiliations:** 1 Department of Animal Sciences and Aquatic Ecology, Faculty of Bioscience Engineering, Ghent University, Coupure Links 653, 9000 Ghent, Belgium; 2 Provincial Centre of Environmental Research, Godshuizenlaan 95, 9000 Ghent, Belgium

**Keywords:** germination, submerged aquatic plants, ecological restoration, lotic ecosystems, nature restoration

## Abstract

Submerged aquatic macrophytes play a key role in stream ecosystems, but their recovery in historically degraded Flemish streams is often limited. This study investigates whether sediment contamination constrains natural macrophyte germination and early seedling establishment. To address this knowledge gap, we combined a controlled mesocosm experiment with an analysis of long-term monitoring data from Flemish streams. The mesocosms showed that higher levels of sediment contamination reduced seedling emergence, indicating that sediment quality can directly inhibit germination and early establishment. In addition, historical monitoring data revealed only a weak association between sediment quality and macrophyte occurrence, pointing to the importance of interacting drivers such as hydrology, light availability, and habitat structure. Together, these findings highlight sediment contamination as a context-dependent but relevant barrier to macrophyte recruitment, underscoring the need to integrate sediment quality into broader restoration planning for streams in Flanders and abroad.

## 1. Introduction

Submerged aquatic macrophytes are vital to stream ecosystem functioning. They improve water clarity, oxygenate the water, absorb excess nutrients, and limit algal blooms [1]. Many species release allelochemicals that suppress algae and cyanobacteria [2,3] and contribute to phytoremediation by accumulating and degrading pollutants [4]. In addition, macrophytes provide a structural habitat that supports diverse aquatic communities [5]. Their presence and composition are therefore widely used as indicators of ecosystem health.

The ecological status of European streams can be assessed not only through chemical parameters but also through biological indicators, including macrophytes, macroinvertebrates, phytoplankton, phytobenthos, and fish [6]. For macrophytes in Flemish streams, the Ecological Quality Coefficient (EQC) rates streams on a scale from 0 (poor) to 1 (reference quality), based on sub-scores for species specificity, disturbance, and growth forms, assessed across three sampling sections [7]. These sub-scores are calculated relative to reference macrophyte conditions defined for each specific waterbody type, and the lowest of the three determines the final EQC [7]. Since 2007, water management interventions have improved overall macrophyte quality, with the proportion of streams classified as “good” rising from under 10% to over 25% [8]. Nonetheless, no streams have yet reached the highest “very good” status, and the majority still fail to meet the ecological threshold for good macrophyte quality [8].

Macrophyte recruitment from the seedbank emerges as a potential factor limiting development [9]. In restored or degraded waterbodies, submerged plant communities must often depend on natural regeneration through seed germination [10]. Successful re-establishment depends not only on favourable light and hydrological conditions but also on the quality of the seed bank [11]. However, environmental stressors such as sediment-associated pollutants may compromise this regeneration potential.

Heavy metal and persistent organic pollutant contamination is known to disrupt early plant development across various species [12]. In terrestrial plants, metals such as cadmium and lead reduce seed viability, delay germination, and impair seedling growth in a concentration- and species-dependent manner [13]. Similarly, pollutants like polycyclic aromatic hydrocarbons (PAHs), polychlorinated biphenyls (PCBs), and C10–C40 hydrocarbons interfere with germination and early development [14,15,16,17]. While these effects are well-documented in terrestrial systems, they remain less thoroughly studied in aquatic environments. Nonetheless, emerging evidence suggests that aquatic and wetland species may be similarly vulnerable. For example, germination experiments with *Triarrhena sacchariflora* and *Phragmites australis* have shown significant reductions in germination rate, germination index, and seedling length under increasing cadmium and mercury concentrations [18].

These concerns are particularly relevant in Flemish streams, where sediment pollution remains a persistent challenge due to historical pollution [19]. Although sediment contamination has generally declined since 2000 in response to improved water quality, many streambeds remain polluted [19,20]. This contrast reflects the long-term persistence of contaminants in sediments, where substances can accumulate and remain bound for decades, even after concentrations in the surface water have declined [21]. According to the most recent biomonitoring cycle (2016–2021), only 31% of stream sediments in Flanders were classified as unpolluted by the Flemish Environment Agency [19]. Consequently, sediment toxicity may continue to constrain vegetation recovery, even in streams where the chemical water quality has improved. Despite growing awareness of this issue, direct experimental evidence linking sediment contamination to macrophyte germination remains scarce [22]. This study addresses this gap by examining how varying levels of sediment contamination affect the natural germination of submerged aquatic macrophytes in streams across Flanders. We hypothesize that (1) higher sediment contamination levels will reduce germination success and seedling establishment, and (2) that these effects will be detectable at both experimental and landscape scales.

To test these hypotheses, two complementary approaches were applied. The first isolates the germination phase in a controlled mesocosm experiment, quantifying germination in stream sediments spanning a gradient of contamination. The second links historical physicochemical scores to long-term and large-scale records of submerged macrophyte coverage across Flanders, with all datasets provided by the Flanders Environmental Agency, to assess whether patterns observed at the experimental scale are reflected in the field. Together, these analyses identify the extent to which sediment pollution limits the natural regeneration of submerged vegetation, providing novel insights into sediment–plant interactions and informing restoration practices.

## 2. Materials and Methods

### 2.1. Mesocosm Experiment

#### 2.1.1. Site Selection and Physicochemical Scoring

**Figure 1:**
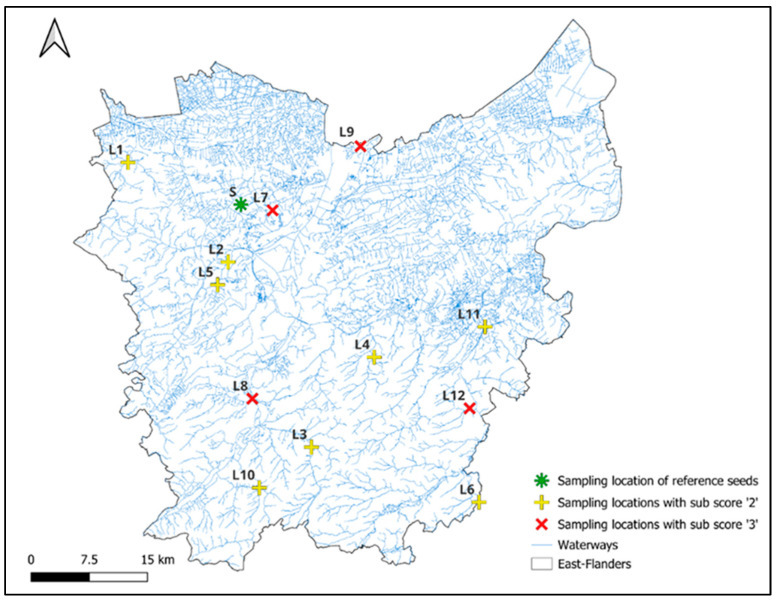
Map of the study area in East-Flanders, Belgium, showing the twelve stream sections selected for sediment sampling in spring 2024, along with their current physicochemical sub-scores. The site where reference seeds were collected for the mesocosm experiment is shown in green. Detailed information on each site is provided in Table 1.

The mesocosm experiment was conducted by collecting sediments from streams in East-Flanders, Belgium. This region includes both lowland streams and moderately sloped streams in the southern Flemish Ardennes. In March 2024, twelve stream sections were sampled across the province for sediment collection and subsequent experimental testing. An overview of all sampled sites is shown in Figure 1; additional details are listed in Table 1.

Site selection was based on historical sediment quality data from the Flemish Environment Agency (VMM). Sediment quality in Flanders is assessed using the Triad Approach [23], which assigns separate sub-scores for the physicochemical, ecotoxicological and biological components of the sediment. Each sub-score ranges from 1 to 4, with higher values indicating poorer quality. For clarity, the scores 1, 2, 3 and 4 are referred to here as “Very Good,” “Good,” “Bad”, and “Very Bad”, respectively. In this study, only the physicochemical sub-score was considered, as it directly reflects sediment characteristics relevant for germination. Additionally, sites were selected on the presence of submerged macrophytes to ensure ecological relevance for germination assessments.

To support interpretation of the experimental results, physicochemical sub-scores were recalculated for each sample location using new quality data from the most recent and newly taken sediment samples. Reliance on historical sub-scores was not an option, as these are only updated in 5-year cycles and would not reliably represent current conditions. The sediment samples of all sites were analysed by the Provincial Centre for Environmental Research (PCM) following the Flemish WACIII protocols, which are the standard procedures for sediment analysis in Flanders [24]. The analysed variables were: clay content (%), organic matter content (%), sum of hydrocarbons C10–C40 (mg/kg DW), sum of pesticides (mg/kg DW), sum of 7 PCBs (mg/kg DW), sum of 6 Borneff PAHs (mg/kg DW), and the individual concentrations of Cd, Cr, Cu, Ni, Pb, Hg, Zn, and As (mg/kg DW). The results are provided in Appendix A. Current physicochemical sub-scores were then calculated from these data using the Triad Approach methodology (Appendix B). This resulted in eight of the sampling sites being classified as “Good” (2) and four sites as “Bad” (3), as shown in Table 1.

#### 2.1.2. Sediment Sampling and Processing

Sediment samples for the laboratory experiment were collected using a coring method commonly employed in lakes (UWITEC gravity corer, Single Corer 60, 6 cm diameter), adapted for shallow streams by omitting weights and the core catcher. The standard WACIII sediment sampling method used by the Flanders Environmental Agency was not applied, because it varies depending on stream characteristics and therefore does not allow direct comparison across sites. By adapting the lake-based coring method for all streams, consistent sample depth and handling were ensured at every location.

At each site, 24 to 30 sub-samples were taken across multiple 1-m-spaced transects perpendicular to the flow, with 3 to 4 cores per transect depending on stream width and substrate conditions. Only the upper 10 cm of sediment, considered the ecologically active layer, was retained. All sub-samples were pooled into one homogenized composite sample per stream.

On the same day of collection, the sediment was sieved (0.5 cm mesh) to remove coarse debris and vegetative material with the potential for vegetative propagation, retaining only naturally occurring seeds. The sieved sediment from each site (*n* = 12) was then used to fill six plastic aquaria (18.5 × 12.2 × 11.8 cm), each with a 4 cm sediment layer (total *n* = 72).

Half of the aquaria per site received 10 reference seeds of *Potamogeton pusillus*, as an addition to naturally occurring seeds, to test for potential seedbank limitation. *P. pusillus* was selected as the reference species because it is one of the common submerged macrophyte species in Flanders, according to monitoring data from the Flemish Environmental Agency, and because it belongs to the genus *Potamogeton*, of which many members are common submerged species in the region. This makes it a suitable representative for a range of widespread submerged macrophyte species in Flanders. In order to procure *P. pusillus* seeds for the seed addition treatment, sediment was collected from the ‘Brakeleike’ stream on the same day as sediment sampling for the aquaria. The collection site was chosen because based on visual observation it contained a dense, naturally occurring population of *P. pusillus*. From approximately 75 L of sediment, 360 seeds were extracted using a 0.5 mm mesh sieve. The identity of all seeds was carefully confirmed using the website https://idtools.org/seed_families (accessed on 20 March 2024), ensuring accurate species identification. Seeds were kept submerged until insertion into the aquaria, with a maximum holding time of four hours.

All aquaria were subsequently filled with 7 cm of tap water and maintained under submerged conditions in a climate-controlled chamber (20 °C, 16:8 h light-dark cycle, fluorescent tube lighting). Air stones were introduced per aquarium for oxygenation. Tap water from the municipal supply was used, which is generally neutral in pH, low in nutrients, and free of detectable pollutants, providing a standard medium for the mesocosm experiments. As a result of this set-up, germination was limited to species able to germinate while fully submerged.

**Figure 2:**
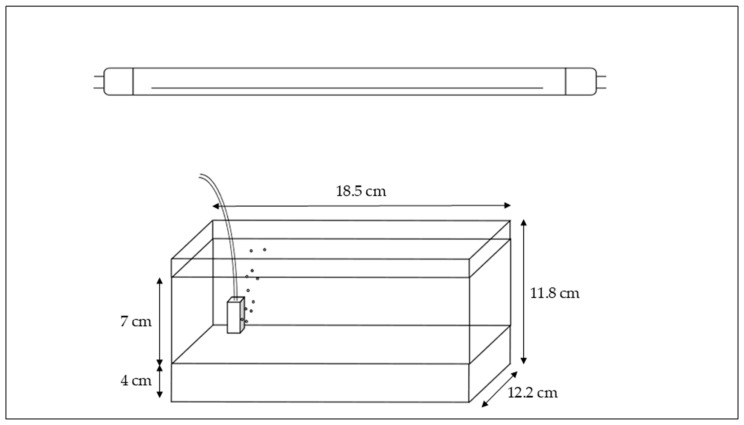
Overview of a single aquarium set-up in the mesocosm experiment, 72 of these aquarium set-ups were included in the experiment. The sediment layer (4 cm) is the bottom layer of the aquarium, and above the water layer (7 cm) with the air stone. The tube connecting the air stone to an air pump is only partly shown in this figure. At the top of the figure, the fluorescent tube lighting is represented as well.

**Figure 3:**
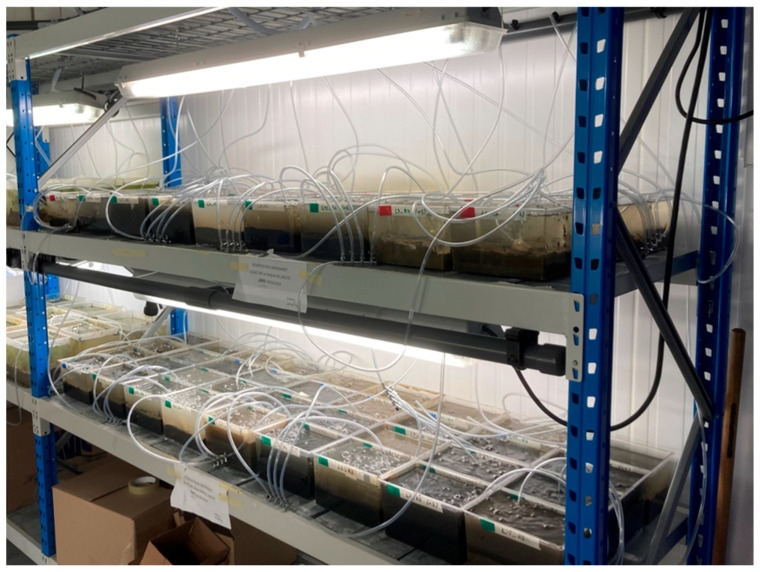
Experimental set-up of 72 aquaria in a climate-controlled chamber.

The interval between field sampling and completing the experimental set-up did not exceed 48 h. During this period, the sediment was kept moist and was stored in a cold room (5 ± 1 °C) overnight. Figure 2 illustrates the set-up for one of the 72 aquaria in the experiment. An image of the complete set-up is provided in Figure 3.

Aquaria placement under the fluorescent tube lighting was randomized at the start of the experiment and reorganized every month. Water levels were checked weekly and replenished with tap water as needed to maintain the 7 cm water level.

#### 2.1.3. Aquaria Monitoring

To monitor the environmental conditions and nutrient dynamics, the following parameters were measured in each aquarium: temperature, pH, dissolved oxygen (DO%), conductivity, turbidity, and chlorophyll *a*. Temperature, pH, DO%, and conductivity were measured using a multiparameter probe (WTW MPP-930, Xylem Analytics, Weilheim, Germany) equipped with three IDS electrodes (Tetracon 925-P for conductivity, SenTix 900-P for pH, and FDO 925-P for DO; all from Xylem Analytics, Weilheim, Germany). All probes included integrated temperature sensors and were calibrated weekly: conductivity with 0.01 M KCl (1278 µS/cm at 20 °C), pH with WTW buffers (pH 7 and pH 4), and DO using water-saturated air. Turbidity and chlorophyll *a* concentrations were measured using an fluorometer/turbidimeter (AquaFluor, Turner Designs, San Jose, CA, USA). Measurements were conducted twice weekly during the first two months, and weekly thereafter to reduce the monitoring effort after conditions had stabilised.

The aquaria were monitored twice each week for seedling emergence, and seedlings were removed immediately to prevent allelopathic interactions. The experiment was conducted over a three-month period, from 20 March to 20 June 2024.

#### 2.1.4. Statistical Analysis Mesocosm Experiment

The effect of sediment quality on germination was assessed using a generalized linear mixed model (GLMM) in R version 4.5.1 with the ‘lme4’ package. The number of germinated seedlings per aquarium was used as the response variable. The sediment quality score (2 or 3) was included as a fixed effect and stream identity as a random intercept to account for non-independence among aquaria from the same site:

Number of Germinations ~ Soil Quality Score + (1|Stream)

Because the response variable represented counts, a Poisson distribution was specified. The model converged successfully without warnings. The variance of the random intercept for stream identity was 0.560 (standard deviation = 0.749), indicating meaningful variability among streams. Overdispersion was low, with a dispersion ratio of 1.14, supporting the appropriateness of the Poisson distribution. Residual diagnostics showed no major deviations from model assumptions. Furthermore, seed addition had no significant effect: reference seeds did not germinate, and total seedling counts were similar in aquaria with and without added seeds. Therefore, seed addition was excluded from the final model.

### 2.2. Historical VMM Data Analysis

The second part of the study used water soil quality data from lotic freshwater ecosystems (streams and rivers) across the entire region of Flanders (Belgium), in contrast to the mesocosm experiment, which was restricted to a subset of sites. All data were obtained from the Flemish Environment Agency (Vlaamse Milieumaatschappij, VMM, Oostende, Belgium) and originated from chemical and ecological field monitoring and analysis. Two primary datasets were extracted: vegetation coverage and sediment physicochemical status.

#### 2.2.1. Vegetation Coverage

Submerged macrophyte coverage was recorded between 1 June 2007, and 5 October 2023, at pre-determined sites for each waterbody. The extent to which sediment surfaces and the water column were colonised was scored on a four-point ordinal scale: 0 = none, 1 = scarce, 2 = frequent to abundant (not filling the water column), and 3 = water column largely filled.

#### 2.2.2. Sediment Physicochemical Status

Sediment contamination was assessed between 1 March 2005 and 17 May 2022 as part of the physicochemical component of the Triad Approach [23]. Scores were categorised into four classes: 1 = Very good, 2 = Good, 3 = Bad, and 4 = Very bad. These are the same types of scores recalculated for the experimental study, enabling direct comparison between experimental and observational findings. Methodology for calculating these sub-scores is provided in Appendix B.

Both datasets included spatial and temporal metadata (coordinates, sampling dates, unique identifiers), which allowed alignment of vegetation and sediment observations.

#### 2.2.3. Data Integration

Biological observations and physicochemical sub-scores were first matched by water body, with duplicates removed to ensure unique vegetation–sediment pairs. Spatial distances between vegetation and sediment sampling points were then calculated using the geosphere package in R (WGS84). For each vegetation observation, the geographically nearest sediment sample within the same water body was retained. If multiple sediment samples were at the same distance, the one with the smallest absolute temporal difference was selected. To ensure ecological relevance, only pairs with a temporal difference of ≤four years (1461 days) were included, reflecting the five-year calculation cycle of physicochemical sediment sub-scores. The final dataset contained 2322 uniquely matched vegetation–sediment pairs that were both spatially proximate and temporally relevant.

#### 2.2.4. Statistical Analysis

Associations between vegetation coverage and sediment physicochemical class were tested using a chi-square test of independence. The effect size was quantified with Cramér’s V to assess the strength of association. All analyses were conducted in R v.4.5.1 using the packages dplyr, geosphere, rcompanion, and forcats. Data were visualised using the ggplot2 package in R, with a bar plot to illustrate the distribution of vegetation coverage across physicochemical classes.

## 3. Results

### 3.1. Physicochemical Sediment Quality Scores

To aid interpretation of the mesocosm experiment, sediment quality scores were derived using the Triad Approach (Appendix B). Each variable was normalized to standard sediment conditions, compared to reference values, and assigned a class (1–4). The overall physicochemical sediment score corresponds to the highest individual class, with a downgrade applied if only one or two variables exceeded the threshold. Table 2 lists the variables that contributed to the highest class and thus (co)determined the final physicochemical score. Raw data for all measured contaminants per sample are listed in Appendix A.

### 3.2. Mesocosm Experiment

**Figure 4:**
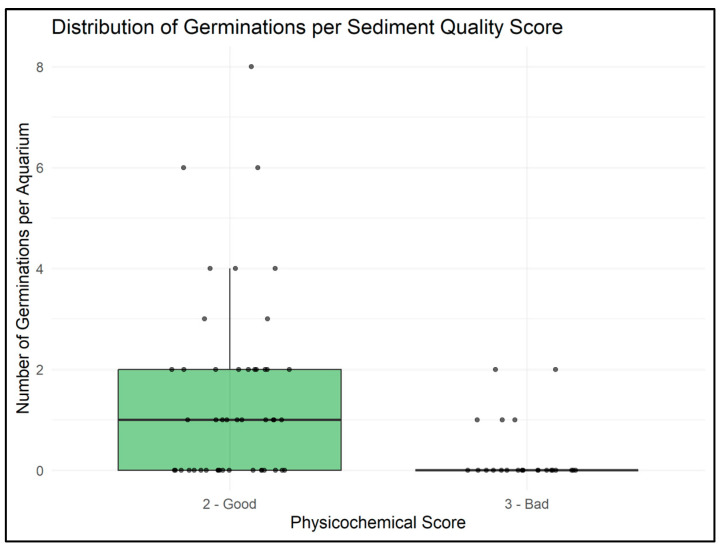
Distribution of germination counts per aquarium for sediments of different quality scores. Sediment quality was classified as 2 (“Good”) or 3 (“Bad”). Boxes show interquartile ranges with medians as horizontal lines; whiskers extend to 1.5× the interquartile range, and points represent individual aquaria. Germination differed significantly between sediment quality classes, with higher counts in sediments of score 2 than in those of score 3 (GLMM, *p* = 0.012).

**Figure 5:**
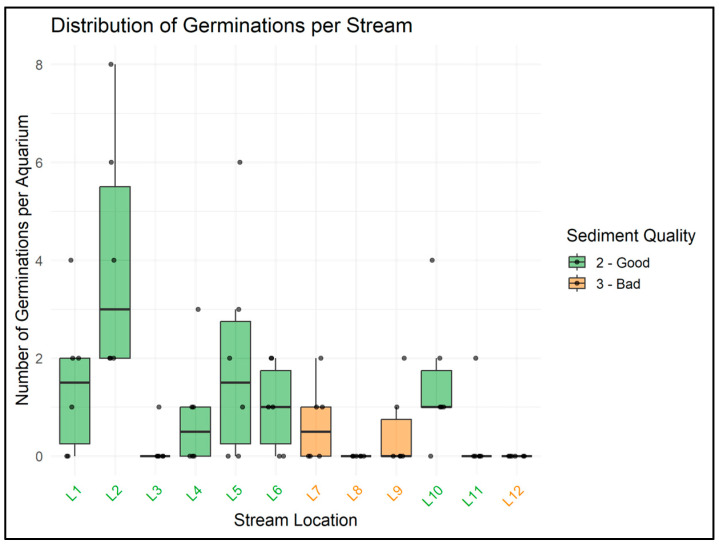
Germination counts per stream, grouped by sediment quality score (2 = Good, 3 = Bad). Boxes indicate interquartile ranges and medians, whiskers extend to 1.5x the interquartile range, and points represent individual aquaria. Although some variation was observed among streams, the overall pattern of lower germination in sediments of lower quality (score 3) was consistent.

Sediment quality significantly affected germination (GLMM, *p* = 0.012). Aquaria with sediments of score 2 (“Good”) showed higher germination counts than those with score 3 (“Bad”) (Figure 4). Pairwise comparisons confirmed the difference between sediment classes. Visual inspection of germination distributions per stream revealed some heterogeneity among streams (Figure 5), but the overall pattern of reduced germination in sediments of lower quality was consistent across sites.

### 3.3. Historical VMM Data

**Figure 6:**
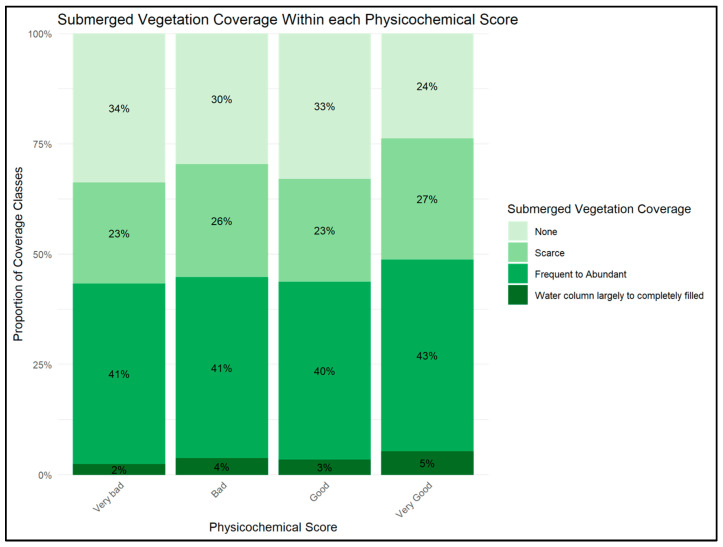
Stacked proportional bar plot showing the relative distribution of submerged vegetation coverage categories across sediment physicochemical classes. Bars are ordered from highest to lowest vegetation coverage, and percentages are annotated for clarity. The distribution of vegetation coverage did not differ significantly across sediment physicochemical classes.

The distribution of vegetation coverage did not differ significantly across sediment physicochemical classes (χ^2^ test of independence, χ^2^ = 12.638, df = 9, *p* = 0.180), and the strength of the association was weak (Cramér’s V = 0.043). Visualization with a stacked proportional bar plot confirmed the lack of a strong pattern. However, the plot hints at a subtle trend: sites with higher sediment quality tended to have a slightly greater proportion of high-coverage observations, whereas sites with lower sediment quality showed a higher proportion of low-coverage observations (Figure 6).

## 4. Discussion

### 4.1. Mesocosm Experiment

Current levels of physicochemical contamination in several Flemish stream sediments inhibit natural macrophyte germination. In our experiment, aquaria containing lower-quality sediments (score 3) produced fewer seedlings than those with higher-quality sediments (score 2), even under optimal experimental conditions. This inhibition may act directly, by interfering with germination processes, or indirectly, by impairing early plant development stages and thus reducing the number of viable seeds available for future generations. These findings are consistent with previous studies showing that heavy metals and persistent organic pollutants reduce seed viability and impair early plant development in both terrestrial and aquatic species [13,18].

Strikingly, in all twelve samples the sum of hydrocarbons C10–C40 contributed to the assigned class, following the Triad Approach methodology (Appendix B). In addition, the sum of the six Borneff PAH’s influenced the classification in three samples, heavy metals (Cu, Cr, Pb, and/or Zn) in two samples, and the sum of seven PCBs in one case. Although the combined effects of different contaminants cannot be disentangled in this experiment, and non-dominant contaminants may also contribute to inhibition, the consistent role of hydrocarbons C10–C40 is notable.

Hydrocarbons in the C10–C40 range, representing medium- to heavy-chain petroleum hydrocarbons, are known to broadly impair seed germination and early plant development, with effects varying across species and contamination levels [17,25,26]. Mechanisms include physical coating of seeds that restricts water and oxygen uptake, as well as toxic effects from hydrocarbon uptake and accumulation [17,27]. Given that the streams in the mesocosm experiment were selected prior to hydrocarbon analysis, the pervasive influence of C10–C40 contamination in East-Flemish sediments highlights an overlooked current barrier to macrophyte restoration.

It should be noted that microbial activity in the sediments may have contributed to the gradual degradation of organic pollutants, including C10–C40 hydrocarbons, which were the main pollutants determining sediment quality scores [28]. While we did not re-measure sediment contamination at the end of the experiment, we expect the impact of this potential degradation on germination results to be limited. The majority of seed germination in all aquaria occurred within the first two weeks, meaning that later changes in pollutant concentrations would have had minimal influence on observed germination differences. Although post-experiment measurements could have provided additional confirmation, the early timing of germination suggests that initial sediment quality was the primary determinant of seed germination.

Consequently, restoration efforts aiming to improve macrophyte coverage should account for sediment contamination when planning reintroductions or habitat improvements. While hydrocarbons C10–C40 were the most consistent contributors to high physicochemical contamination scores, other contaminants, such as Borneff PAHs, heavy metals (Cu, Cr, Pb, Zn), and PCBs, also influenced sediment quality scoring in some samples and may exacerbate inhibitory effects on germination. Reducing or mitigating these sediment contaminant loads may enhance seed viability and germination success, thereby supporting more effective and sustainable macrophyte restoration outcomes.

### 4.2. Historical VMM Data Analysis

Analyses of historical macrophyte coverage and sediment physicochemical scores did not reveal a statistically significant and only a weak association (χ^2^ = 12.638, df = 9, *p* = 0.180; Cramer’s V = 0.043). This finding suggests that, while sediment contamination clearly affected germination in the mesocosm experiment, its effect is not directly reflected in patterns of established macrophyte coverage in the field. Additionally, submerged macrophytes can regenerate through vegetative reproduction and dispersal of vegetative propagules via runoff, which may buffer populations against the effects of sediment contamination on seed germination. Furthermore, the transition from seed germination to sustained vegetation coverage involves multiple ecological stages, including seedling establishment, growth, competition, and survival, each of which may be influenced by additional environmental factors. Consequently, the weak association observed here likely reflects the cumulative influence of hydrology, light availability, flow velocity, and physical habitat structure [29,30], which collectively shape macrophyte assemblages in natural systems. Finally, merging two datasets with differing spatial and temporal resolutions has likely introduced some noise, contributing to a lower effect size. Nevertheless, the visual trend observed in the data suggests that sediment quality may still play a context-dependent role in macrophyte recruitment and persistence, consistent with the patterns identified in the controlled mesocosm experiment.

### 4.3. Integrating Evidence and Implications for Stream Restoration

Combined, our experimental and analytical results indicate that sediment contamination can constrain macrophyte recruitment, but its effects are often overshadowed by other environmental drivers. The mesocosm experiment revealed a clear inhibitory effect of lower-quality sediments on germination under controlled conditions. In contrast, historical field data suggest that this potential is realized variably across streams, likely because other environmental factors, such as water depth, light availability, flow velocity, and physical habitat structure, interact to influence recruitment [29,30].

These findings underscore the importance of assessing sediment quality in restoration planning. In streams with historically contaminated sediments, remediation or removal (e.g., dredging) could enhance germination success and support the establishment of self-sustaining macrophyte populations. However, sediment removal is costly and therefore rarely performed, emphasizing the value of alternative or complementary management approaches, such as improving sediment conditions in situ or prioritizing less contaminated locations.

Importantly, the observed constraints on recruitment indicate that improvements in water quality alone may not immediately translate into increased submerged macrophyte establishment, because sediment recovery is slower and lags behind water quality improvements [21]. In Flemish streams, natural leaching can gradually transfer contaminants from sediments to the water column, as generally sediments are historically more contaminated than the overlying water [19,20]. Provided water quality remains high and no new pollution occurs, leaching can slowly reduce sediment contamination locally over decades, improving conditions for macrophyte establishment. From a restoration perspective, this process is therefore beneficial at the site level, even though mobilized contaminants may be transported downstream and contribute to contaminant loads further along the river network.

Overall, as sediments gradually recover, an increasing number of previously contaminated sites where sediment contamination is currently the primary factor limiting macrophyte recruitment are expected to reach conditions suitable for colation as time progresses. Once established, submerged plants can then further accelerate sediment remediation, as many European species are effective at removing contaminants through phytoremediation. For example, *Callitriche cophocarpa*, *Myriophyllum spicatum*, *Potamogeton crispus*, *Potamogeton pusillus*, and *Potamogeton pectinatus* have been shown to successfully remediate sediments contaminated with heavy metals [31,32,33,34,35], and *P. crispus* has additionally been shown to remediate sediments contaminated with PAHs [36]. As water quality improvements persist over time, sediment quality is thus expected to become an even less dominant limiting factor for macrophyte restoration in these streams.

A limitation of this study is that, following the recalculation of sediment quality classes for consistency across sites, only classes 2 and 3 were represented. However, these classes are representative of current contamination levels, as between 2020 and 2025, over 72% of Flemish streams were classified as score 2 or 3 according to data from the Flemish Environmental Agency. Assessing macrophyte responses across the full spectrum of contamination levels could nevertheless provide a clearer understanding of thresholds and dose-dependent effects. Future research should build on this by investigating longer-term survival and growth under varying contamination levels, examining the effects of specific contaminants in controlled mesocosms, exploring interactions among multiple stressors, and assessing species-specific sensitivity to sediment contamination. Such work would clarify how differential responses shape community composition and ecosystem function, ultimately informing more effective restoration strategies.

## 5. Conclusions

Sediment contamination is a consistent barrier to the germination and early establishment of submerged macrophytes in Flemish streams, with higher contamination reducing recruitment. Mesocosm experiments revealed strong inhibitory effects of contaminated sediments, while historical monitoring data indicate that the effect sizes vary due to interacting factors such as hydrology, light, and habitat structure. For restoration, sites with lower sediment contamination should be prioritized, whereas highly polluted areas may require remediation. By integrating experimental and long-term landscape data, this study provides clear guidance for macrophyte restoration and highlights directions for future research on sediment–plant interactions in historically degraded streams.

## Figures and Tables

**Table 1:** Overview of the sampled stream sites with location details, historical and current physicochemical sub-scores. Current scores were recalculated from recent sediment analyses (PCM, WACIII protocols) using the Triad methodology (Appendix B).

Sample Code	Municipality	Stream	WGS84 X	WGS84 Y	Historical Physicochemical Sub-Score	Current Physicochemical Sub-Score
L1	Maldegem	Biestwatergang	3.430707	51.190528	1	2
L2	Drongen	Kalebeek	3.613135	51.076603	1	2
L3	Zottegem	Zwalmbeek	3.765138	50.863897	1	2
L4	Lede	Molenbeek	3.879407	50.967296	1	2
L5	Deinze	Merebeek	3.594232	51.050456	1	2
L6	Gooik	Papenmeersbeek	4.070531	50.800407	1	2
L7	Evergem	Sleidingsvaardeken	3.694319	51.135785	3	3
L8	Gavere	Moerbeek	3.657409	50.919706	3	3
L9	Wachtebeke	Langelede	3.856119	51.217363	4	3
L10	Maarkedal	Maarkebeek	3.670077	50.817054	3	2
L11	Lebbeke	Oude Dender	4.081488	51.002211	3	2
L12	Denderleeuw	Wildebeek	4.053424	50.908597	4	3

**Table 2:** Variables (co)determining the physicochemical sediment scores by having the highest Triad class in each sample.

Sample Code	Stream	Physicochemical Sediment Score	Contributing Variables
L1	Biestwatergang	2	sum of hydrocarbons C10–C40
L2	Kalebeek	2	sum of hydrocarbons C10–C40
L3	Zwalmbeek	2	sum of hydrocarbons C10–C40
L4	Molenbeek	2	sum of hydrocarbons C10–C40, Cu
L5	Merebeek	2	sum of hydrocarbons C10–C40, sum of 6 PAHs (Borneff), Cr, Cu, Pb, Zn
L6	Papenmeersbeek	2	sum of hydrocarbons C10–C40, sum of 6 PAHs (Borneff)
L7	Sleidingsvaardeken	3	sum of hydrocarbons C10–C40
L8	Moerbeek	3	sum of hydrocarbons C10–C40
L9	Langelede	3	sum of hydrocarbons C10–C40, sum of 6 PAHs (Borneff)
L10	Maarkebeek	2	sum of hydrocarbons C10–C40
L11	Oude Dender	2	sum of hydrocarbons C10–C40
L12	Wildebeek	3	sum of hydrocarbons C10–C40, sum of 7 PCBs

## Data Availability

Data are contained within the article.

## Appendix A. Raw Data, per Site, Used to Recalculate Physicochemical Sub-Scores in Accordance with the Triad Approach ([23])

**Table A1:** Raw data for sampling location L1 (Biestwatergang, Maldegem).

Parameter	Unit	Result
**Soil characteristics**		
Clay content	%	<1.0
Sand content	%	98.5
Organic material content	%	1.40
Dry matter content	%	77.90
Temperature pH measurement	°C	22.80
pH KCl		5.20
**Heavy metals**		
arsenic	mg/kg DW	<4.0
cadmium	mg/kg DW	<0.50
chromium	mg/kg DW	<8.0
copper	mg/kg DW	<7.0
mercury	mg/kg DW	<0.30
lead	mg/kg DW	<10
nickel	mg/kg DW	<4.0
zinc	mg/kg DW	23.00
**Polycyclic aromatic hydrocarbons (PAHs)**		
naphthalene	mg/kg DW	<0.15
acenaphthene	mg/kg DW	<0.10
acenaphthylene	mg/kg DW	<0.10
fluorene	mg/kg DW	<0.10
anthracene	mg/kg DW	<0.10
phenanthrene	mg/kg DW	<0.20
fluoranthene	mg/kg DW	<0.20
pyrene	mg/kg DW	<0.20
chrysene	mg/kg DW	<0.20
benzo(a)anthracene	mg/kg DW	<0.20
benzo(a)pyrene	mg/kg DW	<0.060
benzo(ghi)perylene	mg/kg DW	<0.15
benzo(b)fluoranthene	mg/kg DW	<0.10
benzo(k)fluoranthene	mg/kg DW	<0.10
indeno(1,2,3-cd)pyrene	mg/kg DW	<0.10
dibenzo(a,h)anthracene	mg/kg DW	<0.060
**Polychlorinated biphenyls (PCBs)**		
sum of PCBs	mg/kg DW	<0.014
**Organochlorine pesticides**		
pp-DDT	mg/kg DW	<0.01
op-DDT	mg/kg DW	<0.01
pp-DDE	mg/kg DW	<0.01
op-DDE	mg/kg DW	<0.01
pp-DDD	mg/kg DW	<0.01
op-DDD	mg/kg DW	<0.01
aldrin	mg/kg DW	<0.01
dieldrin	mg/kg DW	<0.01
alpha-HCH	mg/kg DW	<0.01
beta-HCH	mg/kg DW	<0.01
lindane	mg/kg DW	<0.01
cis-chlordane	mg/kg DW	<0.01
trans-chlordane	mg/kg DW	<0.01
alpha-endosulfan	mg/kg DW	<0.05
beta-endosulfan	mg/kg DW	<0.05
endosulfan sulfate	mg/kg DW	<0.01
**Mineral Oils**		
Hydrocarbons (C10–C40)	mg/kg DW	61.00

**Table A2:** Raw data for sampling location L2 (Kalebeek, Drongen).

Parameter	Unit	Result
**Soil characteristics**		
Clay content	%	51.5
Sand content	%	31.8
Organic material content	%	9.70
Dry matter content	%	38.70
Temperature pH measurement	°C	23.50
pH KCl		7.20
**Heavy metals**		
arsenic	mg/kg DW	17.00
cadmium	mg/kg DW	0.89
chromium	mg/kg DW	47.00
copper	mg/kg DW	26.00
mercury	mg/kg DW	<0.30
lead	mg/kg DW	39.00
nickel	mg/kg DW	21.00
zinc	mg/kg DW	125.00
**Polycyclic aromatic hydrocarbons (PAHs)**		
naphthalene	mg/kg DW	<0.15
acenaphthene	mg/kg DW	<0.10
acenaphthylene	mg/kg DW	<0.10
fluorene	mg/kg DW	<0.10
anthracene	mg/kg DW	<0.10
phenanthrene	mg/kg DW	<0.20
fluoranthene	mg/kg DW	<0.20
pyrene	mg/kg DW	<0.20
chrysene	mg/kg DW	<0.20
benzo(a)anthracene	mg/kg DW	<0.20
benzo(a)pyrene	mg/kg DW	<0.060
benzo(ghi)perylene	mg/kg DW	<0.15
benzo(b)fluoranthene	mg/kg DW	<0.10
benzo(k)fluoranthene	mg/kg DW	<0.10
indeno(1,2,3-cd)pyrene	mg/kg DW	<0.10
dibenzo(a,h)anthracene	mg/kg DW	<0.060
**Polychlorinated biphenyls (PCBs)**		
sum of PCBs	mg/kg DW	<0.014
**Organochlorine pesticides**		
pp-DDT	mg/kg DW	<0.01
op-DDT	mg/kg DW	<0.01
pp-DDE	mg/kg DW	<0.01
op-DDE	mg/kg DW	<0.01
pp-DDD	mg/kg DW	<0.01
op-DDD	mg/kg DW	<0.01
aldrin	mg/kg DW	<0.01
dieldrin	mg/kg DW	<0.01
alpha-HCH	mg/kg DW	<0.01
beta-HCH	mg/kg DW	<0.01
lindane	mg/kg DW	<0.01
cis-chlordane	mg/kg DW	<0.01
trans-chlordane	mg/kg DW	<0.01
alpha-endosulfan	mg/kg DW	<0.05
beta-endosulfan	mg/kg DW	<0.05
endosulfan sulfate	mg/kg DW	<0.01
**Mineral Oils**		
Hydrocarbons (C10–C40)	mg/kg DW	295.00

**Table A3:** Raw data for sampling location L3 (Zwalmbeek, Zottegem).

Parameter	Unit	Result
**Soil characteristics**		
Clay content	%	15.8
Sand content	%	72.7
Organic material content	%	3.50
Dry matter content	%	63.80
Temperature pH measurement	°C	23.20
pH KCl		7.30
**Heavy metals**		
arsenic	mg/kg DW	5.60
cadmium	mg/kg DW	<0.50
chromium	mg/kg DW	29.00
copper	mg/kg DW	16.00
mercury	mg/kg DW	<0.30
lead	mg/kg DW	22.00
nickel	mg/kg DW	12.00
zinc	mg/kg DW	80.00
**Polycyclic aromatic hydrocarbons (PAHs)**		
naphthalene	mg/kg DW	<0.15
acenaphthene	mg/kg DW	<0.10
acenaphthylene	mg/kg DW	<0.10
fluorene	mg/kg DW	<0.10
anthracene	mg/kg DW	<0.10
phenanthrene	mg/kg DW	<0.20
fluoranthene	mg/kg DW	<0.20
pyrene	mg/kg DW	<0.20
chrysene	mg/kg DW	<0.20
benzo(a)anthracene	mg/kg DW	<0.20
benzo(a)pyrene	mg/kg DW	<0.060
benzo(ghi)perylene	mg/kg DW	<0.15
benzo(b)fluoranthene	mg/kg DW	<0.10
benzo(k)fluoranthene	mg/kg DW	<0.10
indeno(1,2,3-cd)pyrene	mg/kg DW	<0.10
dibenzo(a,h)anthracene	mg/kg DW	<0.060
**Polychlorinated biphenyls (PCBs)**		
sum of PCBs	mg/kg DW	<0.014
**Organochlorine pesticides**		
pp-DDT	mg/kg DW	<0.01
op-DDT	mg/kg DW	<0.01
pp-DDE	mg/kg DW	<0.01
op-DDE	mg/kg DW	<0.01
pp-DDD	mg/kg DW	<0.01
op-DDD	mg/kg DW	<0.01
aldrin	mg/kg DW	<0.01
dieldrin	mg/kg DW	<0.01
alpha-HCH	mg/kg DW	<0.01
beta-HCH	mg/kg DW	<0.01
lindane	mg/kg DW	<0.01
cis-chlordane	mg/kg DW	<0.01
trans-chlordane	mg/kg DW	<0.01
alpha-endosulfan	mg/kg DW	<0.05
beta-endosulfan	mg/kg DW	<0.05
endosulfan sulfate	mg/kg DW	<0.01
**Mineral Oils**		
Hydrocarbons (C10–C40)	mg/kg DW	150.00

**Table A4:** Raw data for sampling location L4 (Molenbeek, Lede).

Parameter	Unit	Result
**Soil characteristics**		
Clay content	%	20.5
Sand content	%	68.8
Organic material content	%	2.30
Dry matter content	%	70.80
Temperature pH measurement	°C	23.60
pH KCl		7.60
**Heavy metals**		
arsenic	mg/kg DW	<4.0
cadmium	mg/kg DW	<0.50
chromium	mg/kg DW	30.00
copper	mg/kg DW	23.00
mercury	mg/kg DW	<0.30
lead	mg/kg DW	16.00
nickel	mg/kg DW	12.00
zinc	mg/kg DW	42.00
**Polycyclic aromatic hydrocarbons (PAHs)**		
naphthalene	mg/kg DW	<0.15
acenaphthene	mg/kg DW	<0.10
acenaphthylene	mg/kg DW	<0.10
fluorene	mg/kg DW	<0.10
anthracene	mg/kg DW	<0.10
phenanthrene	mg/kg DW	<0.20
fluoranthene	mg/kg DW	<0.20
pyrene	mg/kg DW	<0.20
chrysene	mg/kg DW	<0.20
benzo(a)anthracene	mg/kg DW	<0.20
benzo(a)pyrene	mg/kg DW	<0.060
benzo(ghi)perylene	mg/kg DW	<0.15
benzo(b)fluoranthene	mg/kg DW	<0.10
benzo(k)fluoranthene	mg/kg DW	<0.10
indeno(1,2,3-cd)pyrene	mg/kg DW	<0.10
dibenzo(a,h)anthracene	mg/kg DW	<0.060
**Polychlorinated biphenyls (PCBs)**		
sum of PCBs	mg/kg DW	<0.014
**Organochlorine pesticides**		
pp-DDT	mg/kg DW	<0.01
op-DDT	mg/kg DW	<0.01
pp-DDE	mg/kg DW	<0.01
op-DDE	mg/kg DW	<0.01
pp-DDD	mg/kg DW	<0.01
op-DDD	mg/kg DW	<0.01
aldrin	mg/kg DW	<0.01
dieldrin	mg/kg DW	<0.01
alpha-HCH	mg/kg DW	<0.01
beta-HCH	mg/kg DW	<0.01
lindane	mg/kg DW	<0.01
cis-chlordane	mg/kg DW	<0.01
trans-chlordane	mg/kg DW	<0.01
alpha-endosulfan	mg/kg DW	<0.05
beta-endosulfan	mg/kg DW	<0.05
endosulfan sulfate	mg/kg DW	<0.01
**Mineral Oils**		
Hydrocarbons (C10–C40)	mg/kg DW	70.00

**Table A5:** Raw data for sampling location L5 (Merenbeek, Deinze).

Parameter	Unit	Result
**Soil characteristics**		
Clay content	%	8.8
Sand content	%	88.5
Organic material content	%	3.10
Dry matter content	%	57.80
Temperature pH measurement	°C	23.70
pH KCl		7.80
**Heavy metals**		
arsenic	mg/kg DW	5.70
cadmium	mg/kg DW	<0.50
chromium	mg/kg DW	42.00
copper	mg/kg DW	37.00
mercury	mg/kg DW	<0.30
lead	mg/kg DW	44.00
nickel	mg/kg DW	9.20
zinc	mg/kg DW	166.00
**Polycyclic aromatic hydrocarbons (PAHs)**		
naphthalene	mg/kg DW	<0.15
acenaphthene	mg/kg DW	0.19
acenaphthylene	mg/kg DW	<0.10
fluorene	mg/kg DW	<0.10
anthracene	mg/kg DW	<0.10
phenanthrene	mg/kg DW	<0.20
fluoranthene	mg/kg DW	0.42
pyrene	mg/kg DW	0.28
chrysene	mg/kg DW	<0.20
benzo(a)anthracene	mg/kg DW	<0.20
benzo(a)pyrene	mg/kg DW	0.07
benzo(ghi)perylene	mg/kg DW	<0.15
benzo(b)fluoranthene	mg/kg DW	<0.10
benzo(k)fluoranthene	mg/kg DW	<0.10
indeno(1,2,3-cd)pyrene	mg/kg DW	<0.10
dibenzo(a,h)anthracene	mg/kg DW	<0.060
**Polychlorinated biphenyls (PCBs)**		
sum of PCBs	mg/kg DW	<0.014
**Organochlorine pesticides**		
pp-DDT	mg/kg DW	<0.01
op-DDT	mg/kg DW	<0.01
pp-DDE	mg/kg DW	<0.01
op-DDE	mg/kg DW	<0.01
pp-DDD	mg/kg DW	<0.01
op-DDD	mg/kg DW	<0.01
aldrin	mg/kg DW	<0.01
dieldrin	mg/kg DW	<0.01
alpha-HCH	mg/kg DW	<0.01
beta-HCH	mg/kg DW	<0.01
lindane	mg/kg DW	<0.01
cis-chlordane	mg/kg DW	<0.01
trans-chlordane	mg/kg DW	<0.01
alpha-endosulfan	mg/kg DW	<0.05
beta-endosulfan	mg/kg DW	<0.05
endosulfan sulfate	mg/kg DW	<0.01
**Mineral Oils**		
Hydrocarbons (C10–C40)	mg/kg DW	117.00

**Table A6:** Raw data for sampling location L6 (Papenmeersbeek, Gooik).

Parameter	Unit	Result
**Soil characteristics**		
Clay content	%	18.6
Sand content	%	72.0
Organic material content	%	4.10
Dry matter content	%	65.90
Temperature pH measurement	°C	23.60
pH KCl		7.30
**Heavy metals**		
arsenic	mg/kg DW	8.80
cadmium	mg/kg DW	<0.50
chromium	mg/kg DW	33.00
copper	mg/kg DW	17.00
mercury	mg/kg DW	<0.30
lead	mg/kg DW	32.00
nickel	mg/kg DW	13.00
zinc	mg/kg DW	85.00
**Polycyclic aromatic hydrocarbons (PAHs)**		
naphthalene	mg/kg DW	<0.15
acenaphthene	mg/kg DW	<0.10
acenaphthylene	mg/kg DW	<0.10
fluorene	mg/kg DW	<0.10
anthracene	mg/kg DW	<0.10
phenanthrene	mg/kg DW	<0.20
fluoranthene	mg/kg DW	0.33
pyrene	mg/kg DW	0.27
chrysene	mg/kg DW	0.30
benzo(a)anthracene	mg/kg DW	0.23
benzo(a)pyrene	mg/kg DW	0.28
benzo(ghi)perylene	mg/kg DW	0.22
benzo(b)fluoranthene	mg/kg DW	0.27
benzo(k)fluoranthene	mg/kg DW	0.13
indeno(1,2,3-cd)pyrene	mg/kg DW	0.23
dibenzo(a,h)anthracene	mg/kg DW	0.07
**Polychlorinated biphenyls (PCBs)**		
sum of PCBs	mg/kg DW	<0.014
**Organochlorine pesticides**		
pp-DDT	mg/kg DW	<0.01
op-DDT	mg/kg DW	<0.01
pp-DDE	mg/kg DW	<0.01
op-DDE	mg/kg DW	<0.01
pp-DDD	mg/kg DW	<0.01
op-DDD	mg/kg DW	<0.01
aldrin	mg/kg DW	<0.01
dieldrin	mg/kg DW	<0.01
alpha-HCH	mg/kg DW	<0.01
beta-HCH	mg/kg DW	<0.01
lindane	mg/kg DW	<0.01
cis-chlordane	mg/kg DW	<0.01
trans-chlordane	mg/kg DW	<0.01
alpha-endosulfan	mg/kg DW	<0.05
beta-endosulfan	mg/kg DW	<0.05
endosulfan sulfate	mg/kg DW	<0.01
**Mineral Oils**		
Hydrocarbons (C10–C40)	mg/kg DW	205.00

**Table A7:** Raw data for sampling location L7 (Sleidingsvaardeken, Evergem).

Parameter	Unit	Result
**Soil characteristics**		
Clay content	%	7.1
Sand content	%	88.9
Organic material content	%	6.30
Dry matter content	%	45.20
Temperature pH measurement	°C	23.20
pH KCl		7.40
**Heavy metals**		
arsenic	mg/kg DW	8.80
cadmium	mg/kg DW	0.60
chromium	mg/kg DW	23.00
copper	mg/kg DW	39.00
mercury	mg/kg DW	<0.30
lead	mg/kg DW	49.00
nickel	mg/kg DW	11.00
zinc	mg/kg DW	238.00
**Polycyclic aromatic hydrocarbons (PAHs)**		
naphthalene	mg/kg DW	<0.15
acenaphthene	mg/kg DW	<0.10
acenaphthylene	mg/kg DW	<0.10
fluorene	mg/kg DW	<0.10
anthracene	mg/kg DW	<0.10
phenanthrene	mg/kg DW	<0.20
fluoranthene	mg/kg DW	0.20
pyrene	mg/kg DW	<0.20
chrysene	mg/kg DW	<0.20
benzo(a)anthracene	mg/kg DW	<0.20
benzo(a)pyrene	mg/kg DW	0.09
benzo(ghi)perylene	mg/kg DW	<0.15
benzo(b)fluoranthene	mg/kg DW	0.11
benzo(k)fluoranthene	mg/kg DW	<0.10
indeno(1,2,3-cd)pyrene	mg/kg DW	<0.10
dibenzo(a,h)anthracene	mg/kg DW	<0.060
**Polychlorinated biphenyls (PCBs)**		
sum of PCBs	mg/kg DW	0.016
**Organochlorine pesticides**		
pp-DDT	mg/kg DW	<0.01
op-DDT	mg/kg DW	<0.01
pp-DDE	mg/kg DW	<0.01
op-DDE	mg/kg DW	<0.01
pp-DDD	mg/kg DW	<0.01
op-DDD	mg/kg DW	<0.01
aldrin	mg/kg DW	<0.01
dieldrin	mg/kg DW	<0.01
alpha-HCH	mg/kg DW	<0.01
beta-HCH	mg/kg DW	<0.01
lindane	mg/kg DW	<0.01
cis-chlordane	mg/kg DW	<0.01
trans-chlordane	mg/kg DW	<0.01
alpha-endosulfan	mg/kg DW	<0.05
beta-endosulfan	mg/kg DW	<0.05
endosulfan sulfate	mg/kg DW	<0.01
**Mineral Oils**		
Hydrocarbons (C10–C40)	mg/kg DW	491.00

**Table A8:** Raw data for sampling location L8 (Moerbeek, Gavere).

Parameter	Unit	Result
**Soil characteristics**		
Clay content	%	51.3
Sand content	%	19.3
Organic material content	%	6.50
Dry matter content	%	38.00
Temperature pH measurement	°C	23.20
pH KCl		7.30
**Heavy metals**		
arsenic	mg/kg DW	12.00
cadmium	mg/kg DW	0.67
chromium	mg/kg DW	58.00
copper	mg/kg DW	42.00
mercury	mg/kg DW	<0.30
lead	mg/kg DW	54.00
nickel	mg/kg DW	21.00
zinc	mg/kg DW	223.00
**Polycyclic aromatic hydrocarbons (PAHs)**		
naphthalene	mg/kg DW	<0.15
acenaphthene	mg/kg DW	<0.10
acenaphthylene	mg/kg DW	<0.10
fluorene	mg/kg DW	<0.10
anthracene	mg/kg DW	<0.10
phenanthrene	mg/kg DW	<0.20
fluoranthene	mg/kg DW	0.30
pyrene	mg/kg DW	<0.20
chrysene	mg/kg DW	0.21
benzo(a)anthracene	mg/kg DW	<0.20
benzo(a)pyrene	mg/kg DW	0.13
benzo(ghi)perylene	mg/kg DW	<0.15
benzo(b)fluoranthene	mg/kg DW	0.16
benzo(k)fluoranthene	mg/kg DW	<0.10
indeno(1,2,3-cd)pyrene	mg/kg DW	0.12
dibenzo(a,h)anthracene	mg/kg DW	<0.060
**Polychlorinated biphenyls (PCBs)**		
sum of PCBs	mg/kg DW	<0.014
**Organochlorine pesticides**		
pp-DDT	mg/kg DW	<0.01
op-DDT	mg/kg DW	<0.01
pp-DDE	mg/kg DW	0.014
op-DDE	mg/kg DW	<0.01
pp-DDD	mg/kg DW	<0.01
op-DDD	mg/kg DW	<0.01
aldrin	mg/kg DW	<0.01
dieldrin	mg/kg DW	<0.01
alpha-HCH	mg/kg DW	<0.01
beta-HCH	mg/kg DW	<0.01
lindane	mg/kg DW	<0.01
cis-chlordane	mg/kg DW	<0.01
trans-chlordane	mg/kg DW	<0.01
alpha-endosulfan	mg/kg DW	<0.05
beta-endosulfan	mg/kg DW	<0.05
endosulfan sulfate	mg/kg DW	<0.01
**Mineral Oils**		
Hydrocarbons (C10–C40)	mg/kg DW	526.00

**Table A9:** Raw data for sampling location L9 (Langelede, Wachtebeke).

Parameter	Unit	Result
**Soil characteristics**		
Clay content	%	22.3
Sand content	%	68.1
Organic material content	%	9.70
Dry matter content	%	29.30
Temperature pH measurement	°C	23.10
pH KCl		7.30
**Heavy metals**		
arsenic	mg/kg DW	13.00
cadmium	mg/kg DW	0.92
chromium	mg/kg DW	35.00
copper	mg/kg DW	39.00
mercury	mg/kg DW	0.30
lead	mg/kg DW	43.00
nickel	mg/kg DW	33.00
zinc	mg/kg DW	177.00
**Polycyclic aromatic hydrocarbons (PAHs)**		
naphthalene	mg/kg DW	<0.15
acenaphthene	mg/kg DW	<0.10
acenaphthylene	mg/kg DW	<0.10
fluorene	mg/kg DW	<0.10
anthracene	mg/kg DW	0.12
phenanthrene	mg/kg DW	Interference
fluoranthene	mg/kg DW	1.25
pyrene	mg/kg DW	0.94
chrysene	mg/kg DW	0.79
benzo(a)anthracene	mg/kg DW	0.66
benzo(a)pyrene	mg/kg DW	0.85
benzo(ghi)perylene	mg/kg DW	0.55
benzo(b)fluoranthene	mg/kg DW	0.77
benzo(k)fluoranthene	mg/kg DW	0.39
indeno(1,2,3-cd)pyrene	mg/kg DW	0.61
dibenzo(a,h)anthracene	mg/kg DW	0.18
**Polychlorinated biphenyls (PCBs)**		
sum of PCBs	mg/kg DW	<0.014
**Organochlorine pesticides**		
pp-DDT	mg/kg DW	<0.01
op-DDT	mg/kg DW	<0.01
pp-DDE	mg/kg DW	<0.01
op-DDE	mg/kg DW	<0.01
pp-DDD	mg/kg DW	<0.01
op-DDD	mg/kg DW	<0.01
aldrin	mg/kg DW	<0.01
dieldrin	mg/kg DW	<0.01
alpha-HCH	mg/kg DW	<0.01
beta-HCH	mg/kg DW	<0.01
lindane	mg/kg DW	<0.01
cis-chlordane	mg/kg DW	<0.01
trans-chlordane	mg/kg DW	<0.01
alpha-endosulfan	mg/kg DW	<0.05
beta-endosulfan	mg/kg DW	<0.05
endosulfan sulfate	mg/kg DW	<0.01
**Mineral Oils**		
Hydrocarbons (C10–C40)	mg/kg DW	570.00

**Table A10:** Raw data for sampling location L10 (Maarkebeek, Maarkedal).

Parameter	Unit	Result
**Soil characteristics**		
Clay content	%	16.9
Sand content	%	72.5
Organic material content	%	1.90
Dry matter content	%	72.60
Temperature pH measurement	°C	23.40
pH KCl		7.00
**Heavy metals**		
arsenic	mg/kg DW	5.70
cadmium	mg/kg DW	<0.50
chromium	mg/kg DW	34.00
copper	mg/kg DW	11.00
mercury	mg/kg DW	<0.30
lead	mg/kg DW	17.00
nickel	mg/kg DW	12.00
zinc	mg/kg DW	43.00
**Polycyclic aromatic hydrocarbons (PAHs)**		
naphthalene	mg/kg DW	<0.15
acenaphthene	mg/kg DW	<0.10
acenaphthylene	mg/kg DW	<0.10
fluorene	mg/kg DW	<0.10
anthracene	mg/kg DW	<0.10
phenanthrene	mg/kg DW	<0.20
fluoranthene	mg/kg DW	<0.20
pyrene	mg/kg DW	<0.20
chrysene	mg/kg DW	<0.20
benzo(a)anthracene	mg/kg DW	<0.20
benzo(a)pyrene	mg/kg DW	0.07
benzo(ghi)perylene	mg/kg DW	<0.15
benzo(b)fluoranthene	mg/kg DW	0.11
benzo(k)fluoranthene	mg/kg DW	<0.10
indeno(1,2,3-cd)pyrene	mg/kg DW	<0.10
dibenzo(a,h)anthracene	mg/kg DW	<0.060
**Polychlorinated biphenyls (PCBs)**		
sum of PCBs	mg/kg DW	<0.014
**Organochlorine pesticides**		
pp-DDT	mg/kg DW	<0.01
op-DDT	mg/kg DW	<0.01
pp-DDE	mg/kg DW	<0.01
op-DDE	mg/kg DW	<0.01
pp-DDD	mg/kg DW	<0.01
op-DDD	mg/kg DW	<0.01
aldrin	mg/kg DW	<0.01
dieldrin	mg/kg DW	<0.01
alpha-HCH	mg/kg DW	<0.01
beta-HCH	mg/kg DW	<0.01
lindane	mg/kg DW	<0.01
cis-chlordane	mg/kg DW	<0.01
trans-chlordane	mg/kg DW	<0.01
alpha-endosulfan	mg/kg DW	<0.05
beta-endosulfan	mg/kg DW	<0.05
endosulfan sulfate	mg/kg DW	<0.01
**Mineral Oils**		
Hydrocarbons (C10–C40)	mg/kg DW	73.00

**Table A11:** Raw data for sampling location L11 (Oude Dender, Lebbeke).

Parameter	Unit	Result
**Soil characteristics**		
Clay content	%	37.3
Sand content	%	51.2
Organic material content	%	6.10
Dry matter content	%	47.70
Temperature pH measurement	°C	22.50
pH KCl		7.30
**Heavy metals**		
arsenic	mg/kg DW	8.70
cadmium	mg/kg DW	<0.50
chromium	mg/kg DW	48.00
copper	mg/kg DW	19.00
mercury	mg/kg DW	<0.30
lead	mg/kg DW	19.00
nickel	mg/kg DW	18.00
zinc	mg/kg DW	84.00
**Polycyclic aromatic hydrocarbons (PAHs)**		
naphthalene	mg/kg DW	<0.15
acenaphthene	mg/kg DW	<0.10
acenaphthylene	mg/kg DW	<0.10
fluorene	mg/kg DW	<0.10
anthracene	mg/kg DW	<0.10
phenanthrene	mg/kg DW	<0.20
fluoranthene	mg/kg DW	<0.20
pyrene	mg/kg DW	<0.20
chrysene	mg/kg DW	<0.20
benzo(a)anthracene	mg/kg DW	<0.20
benzo(a)pyrene	mg/kg DW	<0.060
benzo(ghi)perylene	mg/kg DW	<0.15
benzo(b)fluoranthene	mg/kg DW	<0.10
benzo(k)fluoranthene	mg/kg DW	<0.10
indeno(1,2,3-cd)pyrene	mg/kg DW	<0.10
dibenzo(a,h)anthracene	mg/kg DW	<0.060
**Polychlorinated biphenyls (PCBs)**		
sum of PCBs	mg/kg DW	<0.014
**Organochlorine pesticides**		
pp-DDT	mg/kg DW	<0.01
op-DDT	mg/kg DW	<0.01
pp-DDE	mg/kg DW	<0.01
op-DDE	mg/kg DW	<0.01
pp-DDD	mg/kg DW	<0.01
op-DDD	mg/kg DW	<0.01
aldrin	mg/kg DW	<0.01
dieldrin	mg/kg DW	<0.01
alpha-HCH	mg/kg DW	<0.01
beta-HCH	mg/kg DW	<0.01
lindane	mg/kg DW	<0.01
cis-chlordane	mg/kg DW	<0.01
trans-chlordane	mg/kg DW	<0.01
alpha-endosulfan	mg/kg DW	<0.05
beta-endosulfan	mg/kg DW	<0.05
endosulfan sulfate	mg/kg DW	<0.01
**Mineral Oils**		
Hydrocarbons (C10–C40)	mg/kg DW	269.00

**Table A12:** Raw data for sampling location L12 (Wildebeek, DenderLeeuw).

Parameter	Unit	Result
**Soil characteristics**		
Clay content	%	31.3
Sand content	%	53.3
Organic material content	%	4.20
Dry matter content	%	60.50
Temperature pH measurement	°C	23.70
pH KCl		7.60
**Heavy metals**		
arsenic	mg/kg DW	9.60
cadmium	mg/kg DW	<0.50
chromium	mg/kg DW	35.00
copper	mg/kg DW	18.00
mercury	mg/kg DW	<0.30
lead	mg/kg DW	27.00
nickel	mg/kg DW	19.00
zinc	mg/kg DW	97.00
**Polycyclic aromatic hydrocarbons (PAHs)**		
naphthalene	mg/kg DW	<0.15
acenaphthene	mg/kg DW	<0.10
acenaphthylene	mg/kg DW	<0.10
fluorene	mg/kg DW	<0.10
anthracene	mg/kg DW	<0.10
phenanthrene	mg/kg DW	<0.20
fluoranthene	mg/kg DW	<0.20
pyrene	mg/kg DW	<0.20
chrysene	mg/kg DW	<0.20
benzo(a)anthracene	mg/kg DW	<0.20
benzo(a)pyrene	mg/kg DW	0.06
benzo(ghi)perylene	mg/kg DW	<0.15
benzo(b)fluoranthene	mg/kg DW	<0.10
benzo(k)fluoranthene	mg/kg DW	<0.10
indeno(1,2,3-cd)pyrene	mg/kg DW	<0.10
dibenzo(a,h)anthracene	mg/kg DW	<0.060
**Polychlorinated biphenyls (PCBs)**		
sum of PCBs	mg/kg DW	0.090
**Organochlorine pesticides**		
pp-DDT	mg/kg DW	<0.01
op-DDT	mg/kg DW	<0.01
pp-DDE	mg/kg DW	<0.01
op-DDE	mg/kg DW	<0.01
pp-DDD	mg/kg DW	<0.01
op-DDD	mg/kg DW	<0.01
aldrin	mg/kg DW	<0.01
dieldrin	mg/kg DW	<0.01
alpha-HCH	mg/kg DW	<0.01
beta-HCH	mg/kg DW	<0.01
lindane	mg/kg DW	<0.01
cis-chlordane	mg/kg DW	<0.01
trans-chlordane	mg/kg DW	<0.01
alpha-endosulfan	mg/kg DW	<0.05
beta-endosulfan	mg/kg DW	<0.05
endosulfan sulfate	mg/kg DW	<0.01
**Mineral Oils**		
Hydrocarbons (C10–C40)	mg/kg DW	321.00

## Appendix B. Methodology for the Physicochemical Classification of Sediment in Accordance with the Triad Approach ([23])

### Appendix B.1. Variables Considered

The following physicochemical variables are included in the sediment quality classification:

– Clay content (%) and organic matter content (%)
– Sum of hydrocarbons C10–C40
– Sum of pesticides
– Sum of 7 PCBs
– Sum of 6 PAHs (Borneff)
– Heavy metals: Cd, Cr, Cu, Ni, Pb, Hg, Zn, As

### Appendix B.2. Normalization to a Standard Sediment

Measured concentrations in both reference and study sediments are converted to standard sediment conditions (11% clay, 5% organic matter).

For heavy metals:

(A1) $$N\left(11,5\right)=N\left(x,y\right)\frac{A+B\cdot 11+C\cdot 5}{A+B\cdot x+C\cdot y}$$

where N is the concentration at x% clay and y% organic matter, and A, B, C are element-specific constants.

Conversions are only applied within 1–50% clay and 1–20% organic matter. Outside these ranges, boundary values are used.

**Table A13:** Constants for normalization of heavy metals to standard sediment.

Element	A	B	C
Arsenic (As)	10.81	0.10	0.09
Cadmium (Cd)	0.74	0.00	0.005
Chromium (Cr)	24.32	0.72	0.04
Copper (Cu)	27.23	0.22	0.31
Mercury (Hg)	0.20	0.002	0.002
Lead (Pb)	34.72	0.26	0.26
Nickel (Ni)	14.63	0.28	0.12
Zinc (Zn)	196.00	0.50	1.79

For organic contaminants:

N(5) = (5 × N(y))/y, (A2)

where y is the measured organic matter content (%). Conversions are only applied within 1–20% organic matter. Outside these limits, boundary values are used.

### Appendix B.3. Reference Values

For each parameter, a reference concentration is defined as the geometric mean of 12 reference sediments.

**Table A14:** Reference values (geometric mean of 12 reference sites).

Parameter	Reference Value
Arsenic (As)	11 mg/kg DS
Cadmium (Cd)	0.38 mg/kg DS
Chromium (Cr)	17 mg/kg DS
Copper (Cu)	8 mg/kg DS
Mercury (Hg)	0.05 mg/kg DS
Lead (Pb)	14 mg/kg DS
Nickel (Ni)	11 mg/kg DS
Zinc (Zn)	67 mg/kg DS
Sum of hydrocarbons C10–C40	37 mg/kg DS
Sum of pesticides	3.9 µg/kg DS
Sum of 7 PCBs	5.1 µg/kg DS
Sum of 6 PAHs (Borneff)	0.220 mg/kg DS

### Appendix B.4. Ratio to Reference (VTR) and Log Transformation

For each parameter i, the deviation relative to the reference is calculated as:

VTRi = Ns,i(11,5)/Nr,i(11,5), (A3)

where Ns is the normalized concentration of the sample and Nr that of the reference.

Boundary conditions are applied: values < 1 are set equal to 1, and values > 100 are set equal to 100.

The log-transformed index is then calculated:

LogIndexi = log(VTRi), with 0 ≤ LogIndex ≤ 2, (A4)

### Appendix B.5. Classification into Quality Classes

Each parameter is assigned to a class based on LogIndex (Table A15).

**Table A15:** Class assignment per parameter based on LogIndex.

LogIndex	Class	Interpretation
0–<0.4	1	Not deviating
0.4–<0.8	2	Slightly deviating
0.8–<1.2	3	Deviating
1.2–2	4	Strongly deviating

The overall class of a sample corresponds to the highest class observed among parameters. However, a sample will be downgraded by one class if no more than two parameters fall below the midpoint of that class.

## References

[1] Weisner S.E., Strand J.A., Sandsten H. (1997). Mechanisms Regulating Abundance of Submerged Vegetation in Shallow Eutrophic Lakes. Oecologia.

[2] Takeda F., Nakano K., Nishimura O., Shimada Y., Fukuro S., Tanaka H., Hayashi N., Inamori Y. (2011). Allelopathic Potential of *Potamogeton pusillus* Community against *Microcystis aeruginosa*. J. Water Environ. Technol..

[3] Zuo S., Wan K., Ma S., Ye L. (2014). Combined Allelopathic Potential of Aquatic Plants Species to Control Algae. Allelopath. J..

[4] Ansari A.A., Naeem M., Gill S.S., AlZuaibr F.M. (2020). Phytoremediation of Contaminated Waters: An Eco-Friendly Technology Based on Aquatic Macrophytes Application. Egypt. J. Aquat. Res..

[5] Thomaz S.M., Cunha E.R. (2010). The Role of Macrophytes in Habitat Structuring in Aquatic Ecosystems: Methods of Measurement, Causes and Consequences on Animal Assemblages’ Composition and Biodiversity. Acta Limnol. Bras..

[6] European Parliament, Council of the European Union (2000). Water Framework Directive 2000/60/EC. https://eur-lex.europa.eu/resource.html?uri=cellar:5c835afb-2ec6-4577-bdf8-756d3d694eeb.0004.02/DOC_1&format=PDF.

[7] Vlaamse Milieumaatschappij (VMM) (2015). Meetstrategie en Methodiek Macrophyten.

[8] Vlaamse Milieumaatschappij (VMM) (2024). Macrofyten. https://vmm.vlaanderen.be/feiten-cijfers/water/kwaliteit-waterlopen/ecologische-indicatoren/indicator-macrofyten?activeAccordion=d646cdca-31b4-47d6-84e3-2b99c2bb6954%2Cafc7ccf7-bbd6-40cb-9ca3-777d76de259d%2C24754406-3d6b-4855-a104-673bfb8951dd.

[9] Grillas P., Garcia-Murillo P., Geertz-Hansen O. (1993). Submerged Macrophyte Seed Bank in a Mediterranean Temporary Marsh: Abundance and Relationship with Established Vegetation. Oecologia.

[10] Rybak M., Rosinska J., Wejnerowski Ł., Rodrigo M.A., Joniak T. (2024). Submerged Macrophyte Self-Recovery Potential behind Restoration Treatments: Sources of Failure. Front. Plant Sci..

[11] Bakker E.S., Sarneel J.M., Gulati R.D., Liu Z., van Donk E. (2013). Restoring Macrophyte Diversity in Shallow Temperate Lakes: Biotic versus Abiotic Constraints. Hydrobiologia.

[12] Uslu Ö.S., Gedik O., Kaya A.R., Erol A., Babur E., Khan H., Seleiman M.F., Wasonga D.O. (2025). Effects of Different Irrigation Water Sources Contaminated with Heavy Metals on Seed Germination and Seedling Growth of Different Field Crops. Water.

[13] Kranner I., Colville L. (2011). Metals and Seeds: Biochemical and Molecular Implications and Their Significance for Seed Germination. Environ. Exp. Bot..

[14] Yun Y., Liang L., Wei Y., Luo Z., Yuan F., Li G., Sang N. (2019). Exposure to Nitro-PAHs Interferes with Germination and Early Growth of *Hordeum vulgare* via Oxidative Stress. Ecotoxicol. Environ. Saf..

[15] Reddy K.R., Chirakkara R.A., Martins Ribeiro L.F. (2020). Effects of Elevated Concentrations of Co-Existing Heavy Metals and PAHs in Soil on Phytoremediation. J. Hazard. Toxic Radioact. Waste.

[16] Subramanian S., Schnoor J.L., Van Aken B. (2017). Effects of Polychlorinated Biphenyls (PCBs) and Their Hydroxylated Metabolites (OH-PCBs) on *Arabidopsis thaliana*. Environ. Sci. Technol..

[17] Masakorala K., Yao J., Chandankere R., Yuan H., Liu H., Yu C., Cai M. (2013). Effects of Petroleum Hydrocarbon Contaminated Soil on Germination, Metabolism and Early Growth of Green Gram, *Vigna radiata* L. Bull. Environ. Contam. Toxicol..

[18] Peng H., Geng W., Yong-Quan W., Mao-Teng L., Jun X., Long-Jiang Y. (2010). Effect of Heavy Metal Stress on Emerging Plants Community Constructions in Wetland. Water Sci. Technol..

[19] Vlaamse Milieumaatschappij (VMM) Indicator of Water Soil Quality. https://vmm.vlaanderen.be/feiten-cijfers/water/kwaliteit-waterlopen/waterbodem/indicator-waterbodemkwaliteit.

[20] Vlaamse Milieumaatschappij (VMM) (2025). Fysisch-Chemische Toestand van Waterlopen.

[21] Kluska M., Jabłonska J. (2023). Variability and Heavy Metal Pollution Levels in Water and Bottom Sediments of the Liwiec and Muchawka Rivers (Poland). Water.

[22] Ceschin S., Bellini A., Scalici M. (2021). Aquatic plants and ecotoxicological assessment in freshwater ecosystems: A review. Environ. Sci. Pollut. Res..

[23] De Cooman W. Sediment Characterisation of Rivers in Flanders: The Triad Approach. Proceedings of the CATS 4: Characterisation and Treatment of Sediments.

[24] Vlaamse Milieumaatschappij (VMM) (2022). Compendium for Sampling, Measurement, and Analysis of Water and Sediment (WACIII).

[25] El-Tantawy H., El-Hilaly A. (2001). Effect of Petroleum Oil on the Germination, Growth and Yield of Broad Bean Plants. Taeckholmia.

[26] Sorana O.Ț., Mihăilescu S., Strat D., Florentina G.I. (2020). Effects of oil pollution on seed germination and seedling emergence toxicity. Rom. Biotechnol. Lett..

[27] Adam G., Duncan H.J. (2002). Influence of Diesel Fuel on Seed Germination. Environ. Pollut..

[28] Das N., Chandran P. (2011). Microbial degradation of petroleum hydrocarbon contaminants: An overview. Biotechnol. Res. Int..

[29] Dudgeon D., Arthington A.H., Gessner M.O., Kawabata Z.-I., Knowler D.J., Lévêque C., Naiman R.J., Prieur-Richard A.-H., Soto D., Stiassny M.L.J. (2006). Freshwater Biodiversity: Importance, Threats, Status and Conservation Challenges. Biol. Rev..

[30] Riis T., Schultz R., Olsen H.-M., Katborg C.K. (2009). Transplanting Macrophytes to Rehabilitate Streams: Experience and Recommendations. Aquat. Ecol..

[31] Augustynowicz J., Tokarz K., Baran A. (2014). Phytoremediation of Water Polluted by Thallium, Cadmium, Zinc, and Lead with the Use of Macrophyte *Callitriche cophocarpa*. Arch. Environ. Contam. Toxicol..

[32] Norouznia H., Hamidian A.H. (2014). Phytoremediation Efficiency of Pondweed (*Potamogeton crispus*) in Removing Heavy Metals (Cu, Cr, Pb, As and Cd) from Water of Anzali Wetland. Int. J. Aquat. Biol..

[33] Singh M., Rai U.N., Nadeem U., David A.A. (2014). Role of *Potamogeton pectinatus* in Phytoremediation of Metals. Chem. Sci. Rev. Lett..

[34] Li Y., Song Y., Zhang J., Wan Y. (2023). Phytoremediation Competence of Composite Heavy-Metal-Contaminated Sediments by Intercropping *Myriophyllum spicatum* L. with Two Species of Plants. Int. J. Environ. Res. Public Health.

[35] Rivela C.B., Griboff J., Arán D.S., Cortés F.L., Valdés M.E., Harguinteguy C.A., Monferrán M.V. (2024). Single and Combined Phytoextraction of Lead and Cadmium on Submerged Plants *Potamogeton pusillus* L.: Removal, Bioaccumulation Pattern, and Phytotoxicity. Environ. Sci. Pollut. Res..

[36] Liu S., Meng F., Ding Z., Chi J. (2018). Phytoremediation of PAH-Contaminated Sediments with Different Organic Matter Contents by *Potamogeton crispus* L. Int. J. Phytoremediation.

